# Functional health status in subjects after a motor vehicle accident, with emphasis on whiplash associated disorders: design of a descriptive, prospective inception cohort study

**DOI:** 10.1186/1471-2474-9-168

**Published:** 2008-12-19

**Authors:** Maarten A Schmitt, Nico LU van Meeteren, Anton de Wijer, Paul JM Helders, Yolanda van der Graaf

**Affiliations:** 1 SOMT, Institute for Master Education in Manual Therapy, Amersfoort, The Netherlands; 2 Faculty of Medicine, Utrecht University, University Medical Center, Utrecht, the Netherlands; 3 Department of Pediatric Physical Therapy and Clinical Exercise Physiology, Wilhelmina Children's Hospital, University Utrecht, the Netherlands; 4 Department of Oral-Maxillofacial Surgery, Prosthodontics and Special Dental Care, University Medical Center, Utrecht, The Netherlands; 5 Department of Epidemiology and Health Care, Division Julius Centre for Health Sciences and Primary Care Research University Medical Center Utrecht, The Netherlands; 6 Faculty of Medicine and University Medical Center Utrecht University, Utrecht, The Netherlands

## Abstract

**Background:**

The clinical consequences of whiplash injuries resulting from a motor vehicle accident (MVA) are poorly understood. Thereby, there is general lack of research on the development of disability in patients with acute and chronic Whiplash Associated Disorders.

**Methods/Design:**

The objective is to describe the design of an inception cohort study with a 1-year follow-up to determine risk factors for the development of symptoms after a low-impact motor vehicle accident, the prognosis of chronic disability, and costs. Victims of a low-impact motor vehicle accident will be eligible for participation. Participants with a Neck Disability Index (NDI) score of 7 or more will be classified as experiencing post-traumatic neck pain and will enter the experimental group. Participants without complaints (a NDI score less than 7) will enter the reference group. The cohort will be followed up by means of postal questionnaires and physical examinations at baseline, 3 months, 6 months, and 12 months. Recovery from whiplash-associated disorders will be measured in terms of perceived functional health, and employment status (return to work). Life tables will be generated to determine the 1-year prognosis of whiplash-associated disorders, and risk factors and prognostic factors will be assessed using multiple logistic regression analysis.

**Discussion:**

Little is known about the development of symptoms and chronic disability after a whiplash injury. In the clinical setting, it is important to identify those people who are at risk of developing chronic symptoms.

This inception prospective cohort study will provide insight in the influence of risk factors, of the development of functional health problems, and costs in people with whiplash-associated disorders.

## Background

There is general lack of research on the development of disability in patients with acute and chronic WAD. The clinical consequences of whiplash injuries resulting from a motor vehicle accident (MVA) are poorly understood. Although the prognosis of these whiplash-associated disorders (WAD) is generally thought to be favourable, a systematic review by Cote et al (2001) found that prognosis may vary according to the population sampled and the compensation system of the geographical area studied[1]. A large inception cohort study in Quebec showed that 22% of whiplash claimants returned to their usual activities within 1 week, 53% by 1 month, 70% by 3 months, and 97% by 1 year after the accident[2]. Importantly, a proportion of cases (30% in Quebec) did not return to usual activities by 3 months. In a systematic review of prognostic factors, Scholten-Peeters et al. concluded that high initial pain intensity was a significant prognostic factor for persistent (up to 10 years) symptoms[3]. Several factors were of limited prognostic value for functional recovery: physical factors (restricted range of motion, high number of symptoms), psychosocial factors (previous psychological problems), neuropsychological factors (nervousness), and crash-related (e.g. accident on highway) and treatment-related factors (need to resume physiotherapy)[3]. Scholten-Peeters et al. found that older age, female sex, high acute psychological response, angular deformity of the neck, rear-end collision, and compensation were not associated with an adverse prognosis. The best predictors of outcome in a 3-year follow-up study were SF-36 scores for Bodily Pain and Role Emotional, with higher scores being associated with a better outcome[4].

Given the above, it is clear that the consequences of a disorder, such as WAD, will be different in different people, which in turn has consequences for how patients are managed. The goal of rehabilitation is to improve the health status and quality of life of patients by minimizing the intensity and duration of symptoms. The success of rehabilitation in terms of functioning and health is influenced not only by the underlying disorder or condition, but also by personal and environmental factors[5]. A frequently used conceptual framework to measure functioning and health is the International Classification of Functioning, Disability and Health (ICF) [6], which classifies health and health-related domains according to "body structures and functions" and "activities and participation". The ICF also recognizes that outcome may be influenced by factors such as personal factors (e.g., behaviour changes, coping, lifestyle) and environmental factors (e.g., medical care, rehabilitation, physical environment). Health-care expenditure and the demand for health care services have increased significantly in the Netherlands during the last decade[7], and a substantial part of these costs are due to non-traumatic and trauma-based neck pain and WAD[8]. To our knowledge, no recent data are available on costs due to loss of (un)paid productivity and costs associated with medical and non-medical utilization in relation to WAD.

The general lack of research on the development of disability in patients with acute and chronic WAD prompted us to design a study to evaluate factors that play a role in the development of disability in acute and chronic WAD. This is important because the population with chronic WAD is not homogeneous, but includes patients with varying signs, levels of pain and disability, attitudes and beliefs, and from widely divergent social and work backgrounds[8]. Thus the study has three primary aims:

1 To gain insight into the factors that determine the development of disability and chronicity after a low-impact MVA.

2. To determine the natural course of WAD, with respect to prognostic subgroups.

3. To evaluate direct and indirect costs related to WAD.

## Methods/Design

**Figure 1:**
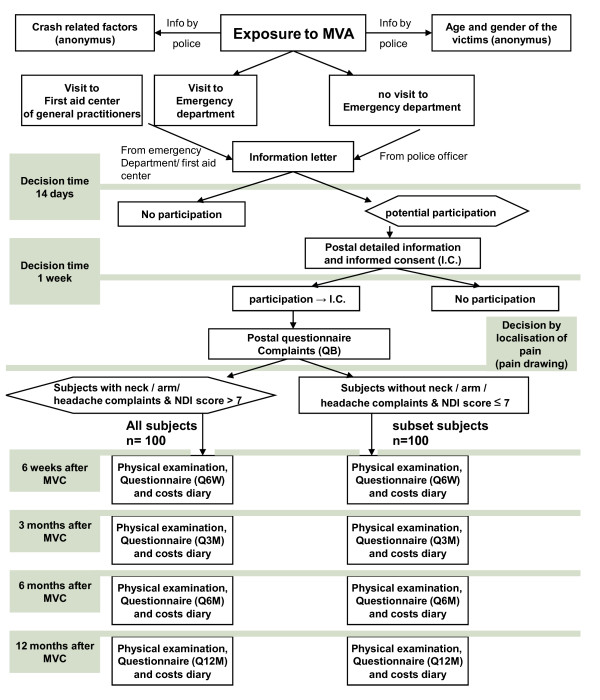
**Study design**. MVA = Motor Vehicle Accident; NDI = Neck Disability Index; QB = first (basic) postal questionnaire; Q6W = Postal questionnaire 6 weeks after enrolment; Q3M = Postal questionnaire 3 months after enrolment; Q6M = Postal questionnaire 6 months after enrolment; Q12M = Postal questionnaire 12 months after enrolment;

This descriptive and prospective inception cohort study will be conducted at the Utrecht Medical Centre. The study was approved by the Ethical Review Board of the University Medical Centre Utrecht. The study consists of two parts, a descriptive study (accident-related factors, functional health status, and costs, and the clinical course of WAD), and a prospective cohort study (prognostic and aetiological factors). The design of this study is presented in Figure 1.

### Study population

Two hundred persons who are exposed to a low-impact MVA will be recruited with the assistance of local police officers, emergency departments of general hospitals, and the emergency medical services. These people will be sent a letter informing them about the study and asking them to participate. Those interested in participating will be asked to reply (by letter, phone or email) within 2 weeks. If they meet the inclusion criteria, they will receive detailed information concerning the aims and procedures of the study and will be asked to give their informed consent.

Participants who have provided informed consent will be sent a postal questionnaire 3 weeks after the MVA. The questionnaire will contain a picture of a blank manikin on which respondents are asked to shade the side on which they experience any pain after the MVA. Participants will also asked to state the intensity of the pain on a numerical pain rating scale (NRS) and to complete the Neck Disability Questionnaire (NDI) Dutch Language Version. On the basis of this information, participants will be divided into two groups: 1] those with pain of the head, neck and/or upper extremity/extremities and an NDI score of more than 7, who will form the experimental group (n = 100), and 2] those who are exposed to a MVA, but without pain in the neck, head, and/or arm (NDI score of 7 or less), and who have no other symptoms, will be selected to form the reference group (n = 100). To form this reference group, every third person without complaints after the MVA will be selected in sequence of time, up to a total number of 100.

#### Inclusion Criteria

Inclusion criteria are involvement in a low-impact MVA (with a speed at the time of the accident less than 50 km/h) 3 weeks before inclusion, no loss of consciousness during and immediately after the accident, and no amnesia after the accident. Subjects have to be able to speak and read Dutch fluently. Subjects must have read the information letter and signed informed consent. Involvement in litigation is not an exclusion criterion but subjects' litigation status will be recorded. Subjects will be allowed to use their current medication and may change medication if necessary.

#### Exclusion Criteria

Exclusion criteria are hospitalization for more than 12 hours after the MVA, fracture or dislocation of the cervical spine and thoracic spine (as determined by X-ray), neurological disorders, head trauma, and inability to read or speak Dutch fluently.

A letter will be sent to each subjects' medical practitioner to inform him/her about the participation of his/her client in the study, together with the results of the screening process. The number of people invited to participate, the number of potential subjects willing to participate, and the number of eligible people will be recorded, as will the reasons for ineligibility. The researchers will have access only to the names and addresses of potential participants before recruitment.

#### Sample size

The annual incidence of whiplash injury in the Netherlands is 94–188 per 100,000 inhabitants. Based on the epidemiological data of Borghouts et al. [7,9] and Picavet et al. [10,11], there are minimally 1060 and maximally 2105 patients with whiplash per year. The number of patients with chronic whiplash syndrome will range from 106–210 (10% of whom develop chronic symptoms) to 530–1052 (50% of whom develop chronic symptoms). The number of MVA victims who do not develop a whiplash syndrome cannot be estimated based on the data of Borghouts[7,9] and Picavet[10,11]. We will use a pre-specified prediction rule, with 7 predictive factors (age, level of activities, neck pain, anxiety, depression, hypochondriasis, and perceived functional health). The sample size calculation is based on the anticipated effect size of 0.2, and 7 predictors. The study is designed to have a statistical power of 80% for the primary comparison. Calculation showed a minimal sample size of 100.

### Baseline Measures

Important determinants and variables of WAD will be evaluated, using validated instruments [12-16].

#### Functional Health Measurement

The Bournemouth Questionnaire for Neck Pain (NBQ)[16] will be used for the measurement of functional health status, in order to describe the natural course of WAD. The NBQ is a perception-based 7-item instrument containing questions relating to pain, physical disability, social disability, anxiety, depression, fear avoidance thoughts in relation to work, and own ability to control pain[16]. The NBQ questionnaire covers impairments, activity limitations, and restrictions in participation. The Neck Disability Index[17] will be used as a perception-based measure of function. Function of the cervical spine will be assessed by means of active range of motion measurement, measurement of deep cervical flexor muscle function according to Falla et al., [18], and evaluation of joint position function. Pain in the neck and arm will be measured with the numeric rating scale for pain (NRS). Physical function will be assessed by measuring lifting capacity[19], as a performance-based measure of general health. Health-related quality of life will be measured using the SF-36. According to Lurie, the SF-36 has the best balance between length, reliability, validity, responsiveness, and experience in large populations of patients with spinal problems[20]. Because participants may perceive themselves to have functional restrictions in a social context, we will include self-report measures of physical functioning and disability such as the work capacity evaluation, and the role limitations scale of the SF-36[21].

#### Measurement of possible mediators

Potential determinants of the development of acute and chronic WAD will be measured. Based on the literature, the following were chosen as being factors that may influence the development of WAD: age, level of activities, neck pain, anxiety and depression, hypochondriasis, and perceived functional health. Recovery from whiplash-associated disorders will be measured in terms of perceived functional health (cut-off point of the sum score of the NBQ: percentage change of scores ≥ 36%)[16], and employment status (return to work). Once participants have been allocated to the experimental or reference group, demographic data, activity level, and employment status will be obtained by means of a questionnaire. The NBQ will be used to measure the perceived functional health. Two measures of employment status will be obtained. A categorical measure of employment status (i.e., working full time, full duties; or working full time, some duties; or working part time, full duties; or working part time, some duties; or employed but not currently working; or employed but not currently working and undergoing re-training; or unemployed, not working) and a continuous measure of employment (i.e., time since change in employment status).

Thus at baseline the following variables will be measured:

• Average pain intensity over last week on a 0–10 scale;

• General symptoms

• Anxiety and depression (Hospital Anxiety Depression Scale)[14]

• Hypochondriasis (Whitely Index)[22]

• Perceived functional health related to neck pain (Neck Bournemouth Questionnaire)[16]

• Activity level (Neck Disability Index)[12].

### Follow-up measurement

The follow-up period will be 1 year, based on the data of Kasch et al. [23], in which inability to work, and no return to normal activities of daily life was seen in 8% of patients with acute WAD patients after 1 year. Outcome data will be collected at 6 weeks, 3 months, 6 months, and 12 months. During the follow-up, physical function will be assessed by a physiotherapist who is unaware of the history of the patients. Other follow-up measures will be obtained by self-report (postal questionnaires).

Effort will be taken to ensure that the protocol is consistently applied. Protocol manuals will be developed and staff will be trained to ensure that screening and assessment are conducted according to the protocol.

If subjects are concerned about their condition during the study, the physiotherapist will screen for potentially serious pathology and, where appropriate, refer participants to a medical practitioner. Subjects will be free to seek any treatment. Subjects will be requested to formally record the type and amount of treatment they receive.

### Data analysis

Descriptive statistics will be used to describe clinical characteristics, demographic characteristics, and psychological, and clinical factors (impairments, functional limitations, and disability). Central estimators of all relevant variables will be calculated. Differences in variables measured at baseline and during follow-up will be analysed with a paired Wilcoxon test. Direct and indirect costs will be calculated based on the estimated number of days that patients are not able to work. The iMTA guideline of cost analysis will be used in the data analysis[24,25]. The main purpose of the prospective part of the study is to describe which factors predict the development of symptoms, impairments, and disability after a MVA. As a first step we will describe univariate relationships between candidate predictors and the outcome (symptoms, impairments, and disability). Only highly likely predictors will be included in this process. All variables with an association with a p-value < 0.20 (Chi-square) will be included in the multivariate analysis. Multivariate analysis will be carried out with multiple logistic regression analysis. The final model will be corrected for overoptimism by shrinking the coefficient. The performance of the model will be investigated with ROC-analysis. The calibration of the model will be tested with the Hosmer Lemeshow test[26]. The final result will be a simple prediction rule. All calculations will be done using SPSS 16.0 software.

## Discussion

This article outlines the rationale and design of a prospective cohort study of risk factors and prognostic factors for WAD, assessed over 1 year. People who have sustained a whiplash injury may develop symptoms, collectively referred to as WAD. However, little is known about which people develop symptoms and become chronically disabled as a result of these disorders[27,28]. Several factors may explain the transition from post-traumatic impairments to functional limitations and restriction of participation[1,3], but these do not entirely explain the development of chronic WAD after whiplash injury [29-32]. In the clinical setting, it is important to identify those people who are risk of developing chronic symptoms as early as possible. By measuring variables within 3 weeks of the MVA, it is hoped that we will be able to identify factors prognostic of the development of WAD after a whiplash injury.

Although the pathophysiology of WAD remains unclear, it is important to identify risk factors for acute and chronic WAD. Despite extensive research, the prognostic factors that play a role in the development of functional health problems, i.e. disability, have not been identified. The ICF is a suitable model to investigate the natural course of WAD and potential prognostic factors. As WAD is a complex, multifaceted problem, it is important that diagnostic studies focus on all the facets that may play a role in the development of chronic WAD. We have presented the rationale and design of an inception prospective cohort study that investigates the influence of risk factors, internal factors, and external factors of the development of functional health problems, and costs in people with whiplash-associated disorders.

## Competing interests

The authors declare that they have no competing interests.

## Authors' contributions

All authors participated in the design of the study. MS drafted the manuscript with input from the other authors. All authors read, revised and approved the final manuscript.

## Pre-publication history

The pre-publication history for this paper can be accessed here:


